# Genetic divergence between reservoir and pre-therapy plasma HIV-1 Env variants does not confer consistent compartment-specific differences in broadly neutralizing antibody sensitivity

**DOI:** 10.1128/jvi.01151-26

**Published:** 2026-09-11

**Authors:** Siyamthemba Dlamini, Doty Ojwach, Jennifer Giandhari, Bongiwe Ndlovu, Zaza Ndhlovu, Thumbi Ndung’u, Jaclyn Mann

**Affiliations:** 1 HIV Pathogenesis Programme, Doris Duke Medical Research Institute, Nelson R. Mandela School of Medicine, University of KwaZulu-Natal 72753, Durban, South Africa; 2 KwaZulu-Natal Research Innovation and Sequencing Platform (KRISP), University of KwaZulu-Natal 706629 , Durban, South Africa; 3 Africa Health Research Institute (AHRI) 145749, Durban, South Africa; 4 Ragon Institute of Massachusetts General Hospital, Massachusetts Institute of Technology, and Harvard University 2167, Cambridge, Massachusetts, USA; 5 Division of Infection and Immunity, University College London 308348, London, United Kingdom; The Ohio State University, Columbus, Ohio, USA

**Keywords:** HIV-1 envelope, HIV-1 subtype C, HIV-1 reservoir, lymph nodes, peripheral blood mononuclear cells, broadly neutralizing antibodies, neutralization assays

## Abstract

Broadly neutralizing antibodies (bNAbs) are a promising strategy to target the HIV-1 reservoir, the main barrier to cure. HIV-1 variants may differ in Env sequence and neutralization sensitivity across anatomical compartments. We investigated genetic differences and differences in neutralization sensitivity between Env variants persisting in peripheral blood mononuclear cell (PBMC) and lymph node (LN) reservoirs, and pre-therapy plasma variants. The study included 11 individuals on antiretroviral therapy in Durban, South Africa. Env sequences derived from pre-therapy plasma and from PBMCs and LN reservoirs were assessed for reservoir-specific genetic differences. Recombinant viruses encoding Env variants were generated for six participants and tested for neutralization sensitivity using a panel of nine clinically relevant bNAbs. We observed genetic divergence between compartments, with a median of 6 and 19 amino acid differences between pre-therapy plasma and LN and PBMC reservoirs, respectively. Of these, 63% (LN) and 73% (PBMC) were confirmed by next-generation sequencing to be unique to the reservoir, with few occurring at sites previously associated with altered bNAb sensitivity. Phenotypic analysis revealed no consistent compartment-specific patterns in neutralization sensitivity. CD4-binding site (CD4bs) and gp120–gp41 interface bNAbs showed broad coverage across compartments, while sensitivity to V2 and V3 antibodies was more variable. Although reservoir-derived Env variants show genetic divergence from pre-therapy viruses, this variation is largely not at sites reported to alter bNAb sensitivity and does not translate into consistent compartment-specific differences in bNAb sensitivity. The broad activity of CD4bs and interface-targeting bNAbs across compartments supports their suitability in antibody-based HIV cure strategies.

**IMPORTANCE:**

HIV persists in reservoirs during antiretroviral therapy, preventing a cure. Broadly neutralizing antibodies (bNAbs) are a promising strategy to target these reservoirs, but it is unclear whether viruses in different tissues respond differently to these antibodies. Here, we show that although HIV variants in blood and lymph node reservoirs differ genetically from viruses present before treatment; they do not show consistent changes in antibody sensitivity. Importantly, antibodies targeting the CD4-binding site and the gp120–gp41 interface remained broadly effective across all compartments. These findings suggest that key bNAbs can target HIV reservoirs throughout different body compartments, supporting their use in cure strategies and highlighting the value of combining antibodies to overcome viral diversity.

## INTRODUCTION

The development of a curative or remission-based strategy for HIV-1 faces a central, unresolved challenge: the persistence of a genetically diverse and stable viral reservoir that evades antiretroviral therapy (ART) and immune clearance (1). One major approach towards HIV cure combines reactivating latently infected cells with eliminating both the virus and reactivated cells through immune-enhancing strategies. Broadly neutralizing antibodies (bNAbs) are a promising immunotherapeutic tool in such a strategy (2). In addition to preventing the infection of new cells, bNAbs can facilitate the clearance of infected cells expressing HIV Env on their surface (3). However, their efficacy is limited by the genetic diversity of Env and the heterogeneous nature of the reservoir (4).

Previous studies have demonstrated that Env variants can differ across anatomical compartments (5), raising the possibility that reservoir variants could exhibit altered susceptibility to neutralizing antibodies. However, limited and conflicting evidence exists regarding compartment-specific patterns in neutralization sensitivity of HIV-1 Env variants. In one study of subtype C viruses derived from plasma, PBMCs, and lymph nodes, partial Env compartmentalization was observed and was partly attributed to mutations that were previously reported to alter sensitivity to bNAbs, although neutralization sensitivity was not directly measured, and most participants were not on ART (6). While Stefic et al. (7) observed Env compartmentalization in the cerebrospinal fluid (CSF) relative to peripheral blood, they found no difference in sensitivity to autologous neutralization between blood- and CSF-derived variants; however, bNAb sensitivity was not assessed, and participants were not on ART (7). In another investigation using computational analysis of Env sequences from multiple tissues collected at autopsy from individuals (most were ART-suppressed at death), the mean neutralization sensitivities for each antibody were similar between compartments, except for some antibodies showing altered sensitivity in the central nervous system (CNS); functional neutralization assays were not performed, and the analysis was limited to subtype B infection (8). On the other hand, a study of paired blood and lymph node samples from subtype B-infected individuals on long-term suppressive ART found little evidence of Env compartmentalization in the reservoir, although neutralization sensitivity was not assessed (9). Taken together, it remains unclear whether reservoir-derived Env variants in different compartments differ in their susceptibility to bNAbs, particularly in ART-suppressed individuals infected with subtype C—the predominant subtype in sub-Saharan Africa.

To address this gap, we investigated whether Env variants persisting in lymph node and PBMC reservoirs differ genetically from pre-therapy plasma variants, and whether these compartment-derived variants exhibit differences in neutralization sensitivity to bNAbs. Env sequences from PL, PBMCs, and LN samples were characterized, and for a subset of participants, replication-competent viruses encoding patient-derived Env were generated to directly assess their sensitivity to a panel of clinically relevant bNAbs.

## MATERIALS AND METHODS

### Study participants

The study cohort comprised eleven participants living with HIV-1 receiving ART, who were enrolled in two Durban-based observational cohorts where participants are followed from acute infection; namely, the Females Rising through Education, Support, and Health (FRESH) cohort (BF131/11) and the HPP Acute Infection Cohort (BE102/14) (10, 11). Five participants initiated ART during early infection, whereas six commenced therapy during the chronic stage of infection. Samples analyzed in this study included plasma collected at or near the time of ART initiation, hereafter referred to as pre-therapy plasma (PL), and post-therapy lymph nodes (LNs) and PBMCs (Table 1). The LNs were voluntarily obtained under an approved LN study protocol (BF298/14). Participants were virally suppressed on ART at the time of LN and PBMC sampling.

**TABLE 1:** Samples from lymph nodes, peripheral blood mononuclear cells (PBMCs), and plasma

Participant ID	Treatment initiation	Treatment date	Plasma date	Lymph node date	PBMC date
594	Early	08/05/15	08/05/15	21/09/16	13/12/16
651	Early	09/08/15	09/08/15	30/11/16	29/09/16
683	Early	29/07/15	11/08/15	29/09/16	05/12/16
714	Early	21/09/15	28/09/15	29/10/16	10/11/16
1210	Early	09/05/17	09/05/17	06/09/17	16/10/17
093	Chronic	05/03/14	06/01/14	02/02/17	23/03/17
1012	Chronic	26/08/16	26/08/16	25/09/18	14/09/18
0039	Chronic	10/03/15	05/03/15	12/04/17	03/04/17
0037	Chronic	22/09/15	28/10/15	29/03/17	23/03/17
0098	Chronic	02/09/16	26/08/16	27/03/18	27/03/18
3830	Chronic	22/10/12	30/08/12	21/06/18	03/04/18

### HIV-1 *env* amplification

Viral RNA was extracted from plasma using the QIAamp Viral RNA Mini Kit (Qiagen). Genomic DNA was isolated from 1 to 2 million LN cells using the MasterPure Complete DNA and RNA Extraction Kit (Lucigen), and from 5 million PBMCs using the DNeasy Blood and Tissue Kit (Qiagen).

As previously described (10), RNA was reverse transcribed into cDNA using SuperScript IV reverse transcriptase (Invitrogen) and an *env*-specific primer, followed by full-length *env* amplification via a nested PCR approach.

### Env sequencing and sequence analysis

Bulk *env* PCR products were sequenced using the BigDye Terminator v3.1 kit (Applied Biosystems) on an ABI 3130xl genetic analyzer. Sequences were edited using Sequencher version 5.4.6 (Gene Codes, Ann Arbor, MI). Env sequences derived from PL, PBMCs, and LNs were aligned using GeneCutter (Los Alamos HIV Database), and amino acid differences between compartments were examined using BioEdit. Insertions relative to the HXB2 reference were removed to ensure consistent codon numbering. For defective reservoir sequences with premature stop codons, APOBEC-associated hypermutation was assessed using the Hypermut tool available through the Los Alamos HIV Database. A sequence was classified as hypermutated if it showed a statistically significant enrichment of APOBEC-associated G to A mutations relative to the corresponding pre-therapy PL sequence, as determined by the Fisher’s exact test (*P* < 0.05). Maximum-likelihood phylogenetic trees were constructed using PhyML (Los Alamos HIV Database) to assess sequence relatedness and confirm participant-specific clustering (12). A subtype C consensus sequence (2021) from the Los Alamos HIV Database was included as a reference.

Next-generation sequencing was performed on bulk pre-therapy PL *env* amplicons using the Illumina MiSeq platform (median coverage: 1,415×) to determine whether mutations identified in reservoir sequences by Sanger sequencing were unique to the reservoir or present in PL at low frequencies prior to therapy initiation. Variants present at frequencies ≥ 2% were detected using Geneious Prime. Mutations unique to reservoir sequences were compared with previously reported Env sites associated with altered bNAb neutralization sensitivity (Table S1) (13) to assess their potential impact on neutralization sensitivity.

### Env recombinant virus generation

Recombinant viruses encoding participant-derived *env* sequences were generated by homologous recombination between an *env* PCR product and a gp160-deleted NL4-3 backbone, based on methods previously described (14). The gp160-deleted NL4-3 backbone, in which NL4-3 nucleotides 5,586–8,184 were deleted and replaced with a BstEII site, was kindly provided by Dr. Zabrina Brumme (Simon Fraser University).

The *env* PCR products were re-amplified using 100-mer primers complementary to NL4-3 sequences flanking the deleted region. The resulting *env* PCR products and BstEII-linearized gp160-deleted NL4-3 plasmid were co-transfected into HEK293T cells to generate recombinant virus. HEK293T cells were seeded in six-well plates and transfected the following day with 1 µg of linearized plasmid and 0.5 µg of *env* PCR product using 8 µL of 1 mg/mL polyethylenimine (PEI). Supernatants containing recombinant virus were harvested at 48 h post-transfection and stored at –80°C. To increase viral titers, HEK293T-derived virus stocks were passaged in GXR25 cells, a CEM-derived T-cell line that expresses GFP upon HIV infection (15). Infection was monitored by flow cytometry, and virus stocks were harvested when approximately 30% of cells were GFP-positive, aliquoted, and stored at –80°C for subsequent neutralization assays. A subset of recombinant virus stocks was partially resequenced to confirm virus identity and assess whether substantial Env sequence changes had occurred during recombinant virus generation and propagation.

### Determining the infectious dose of virus stocks

The infectivity of each recombinant virus stock was determined prior to neutralization assays using TZM-bl cells, as previously described (13). Briefly, TZM-bl cells supplemented with DEAE-dextran were infected with threefold serial dilutions of virus stocks in 96-well plates and incubated at 37°C with 5% CO_2_. After 48 h, culture medium was removed and replaced with Bright-Glo Luciferase Assay Reagent (Promega) and luminescence (relative light units [RLUs]) was measured using a PerkinElmer Victor Nivo luminometer. Virus stocks were diluted in growth media to achieve an infectious dose corresponding to 20,000-60,000 RLUs for neutralization assays.

### Neutralization assay and analysis

Neutralization sensitivity was measured by reduction in luciferase activity following infection of TZM-bl cells with viruses pre-incubated with broadly neutralizing antibodies (bNAbs) (13). A panel of nine broadly neutralizing antibodies (bNAbs) targeting five major epitope regions was evaluated: CD4-binding site (VRC01, VRC07-523LS, N6), V2 apex (CAP256-VRC26.25, PGDM1400), V3-glycan supersite (PGT121, 10-1074), membrane-proximal external region (10E8v4), and gp120–gp41 interface (PGT151). Threefold serial dilutions of each bNAb were prepared in 96-well plates, with starting and final concentrations ranging from 20 to 0.009 µg/mL. Recombinant virus stocks (equivalent to 20,000-60,000 RLUs) were added to antibody-containing wells and incubated for 1 h to allow neutralization. TZM-bl cells (1 × 10⁴/well) supplemented with DEAE-dextran were then added, and luminescence was measured after 48 h, as described above. Virus control (virus only) and cell control (cells only) wells were included in each assay. Neutralization curves were generated by plotting percent neutralization against log₁₀ antibody concentration, and 50% inhibitory concentrations (IC50) were determined by non-linear regression (sigmoidal dose-response) using Microsoft Excel. All assays were performed in duplicate, and mean IC50 values were reported. Neutralization IC50 values were compared across compartments (PL, PBMC reservoir, and LN reservoir) for each antibody. Pairwise comparisons between two compartments at a time were performed using the Wilcoxon signed rank test.

## RESULTS

### Genetic differences between reservoir-derived and pre-therapy plasma Env sequences: few differences at known bNAb escape-associated sites

**Fig 1:**
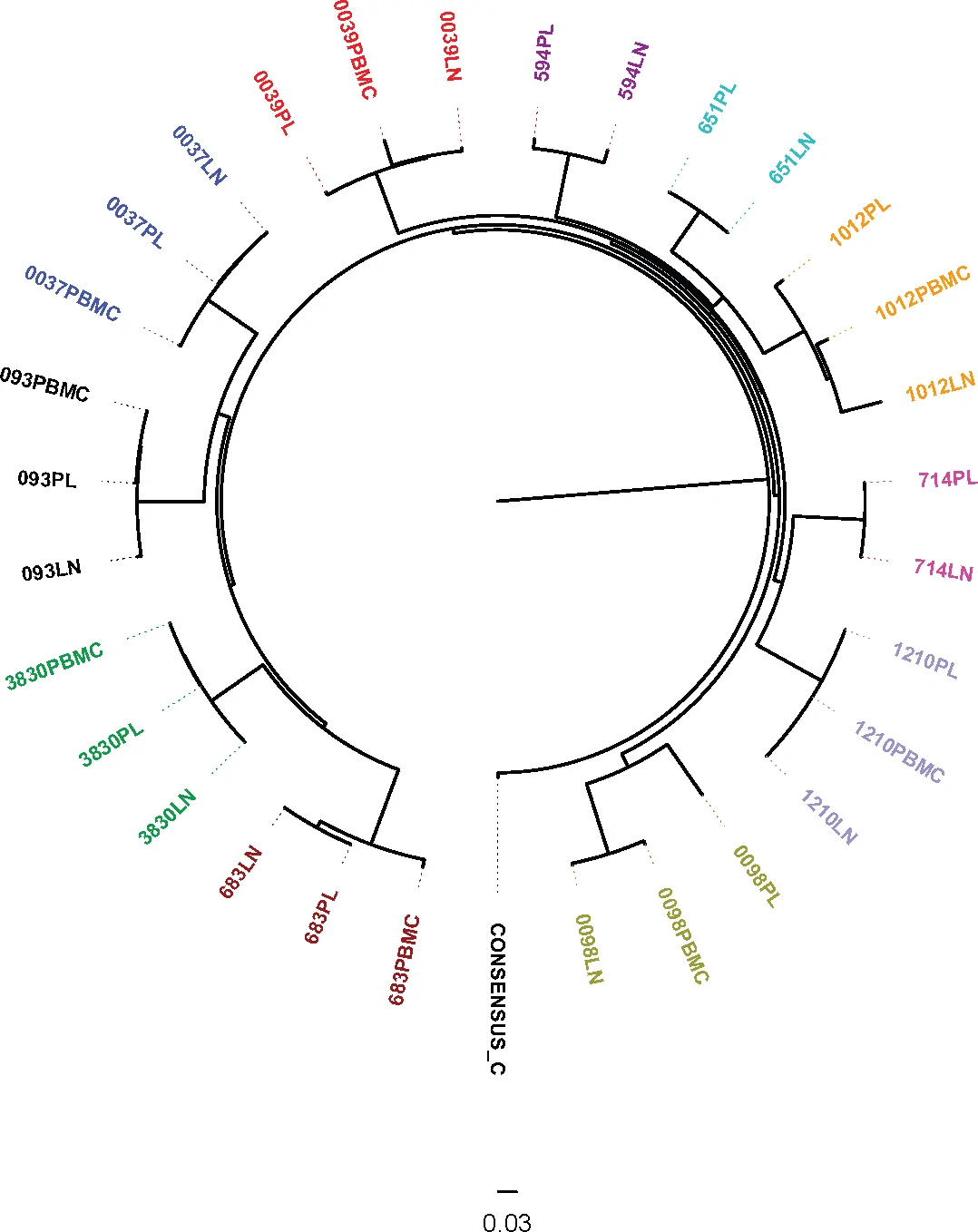
Phylogenetic relatedness of HIV-1 *env* sequences from pre-therapy plasma and post-therapy reservoirs. A maximum-likelihood phylogenetic tree was constructed using PhyML from 30 full-length *env* bulk nucleotide sequences derived from 11 study participants. The pre-therapy PL, PBMC, and LN sequences are color-coded by participant to illustrate their origin. The HIV-1 subtype C consensus sequence from 2021 (www.hiv.lanl.gov) was included as a reference.

We characterized the HIV-1 Env sequences from pre-therapy PL and post-therapy reservoirs from 11 study participants. Amplification of *env* was successful for all samples, except for the PBMC compartments of early-treated participants 594, 651, and 714. A maximum-likelihood phylogenetic tree of *env* sequences confirmed participant-specific clustering (Fig. 1). In addition, it was observed that two sequences from the reservoir (0039 PBMC and 1012 LN) showed evidence of APOBEC-associated hypermutation, as confirmed by the Hypermut tool in the LANL database, and these were therefore excluded from downstream genetic and functional comparisons.

**Fig 2:**
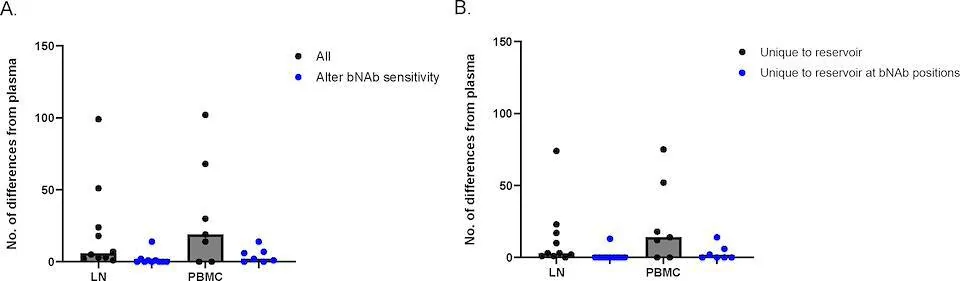
Genetic differences between reservoir-derived and pre-therapy plasma Env sequences. (**A**) The graph shows the average number of amino acid differences between the pre-therapy PL Env sequence and sequences derived from the LN and PBMC reservoirs after at least 1 year of ART (black). A subset of these differences was further analyzed to identify those occurring at known bNAb positions (blue), representing amino acid positions previously reported to alter sensitivity to bNAbs. (**B**) The graph quantifies the total number of unique amino acid mutations in the reservoir sequences (black), as determined by next-generation sequencing of the pre-therapy PL virus, and the subset of these unique mutations occurring at known bNAb sites (blue). Bars indicate the medians.

We observed measurable genetic differences in Env, with a median of 6 (interquartile range [IQR]: 3–22.5) and 19 (IQR: 7–49) Env amino acid differences between pre-therapy PL and the LN and PBMC reservoirs, respectively (Fig. 2A). Next-generation sequencing revealed that 63% (median = 3 mutations per participant, IQR = 1.25–15.25) of the amino acid differences identified between pre-therapy PL and LN sequences, and 73% (median = 14, IQR = 6–35) of those identified between PL and PBMC sequences, were unique to the reservoir and were confirmed by Illumina MiSeq analysis to be absent (<2% frequency) from the corresponding pre-therapy PL population (Fig. 2B). Notably, very few (median = 0 for both LN and PBMC) of the unique reservoir mutations occurred at amino acid positions known to affect bNAb sensitivity (Fig. 2A and B; Table S2).

### Neutralization sensitivity varied by participant and antibody, without consistent compartment-specific patterns

**Fig 3:**
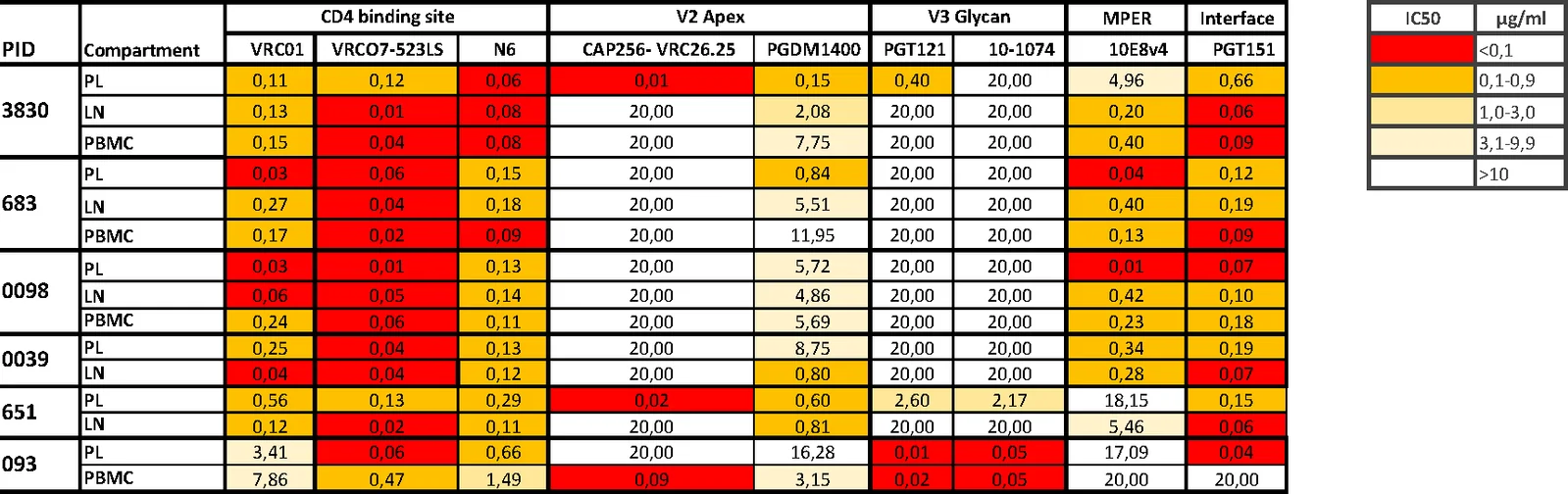
Neutralization sensitivity of the 15 recombinant viruses to nine broadly neutralizing antibodies. A heatmap depicting the 50% inhibitory concentration (IC50) values for recombinant viruses encoding *env* sequences from PL, PBMCs, and LN reservoirs tested against a panel of nine bNAbs. Antibodies are grouped by their target epitope: CD4bs (VRC01, VRC07-523LS, N6), V2 apex (CAP256-VRC26.25, PGDM1400), V3-glycan supersite (PGT121, 10-1074), membrane-proximal external region (10E8v4), and gp120–gp41 interface (PGT151). The color scale represents the degree of sensitivity, with darker shades (red) indicating higher potency (lower IC50, greater sensitivity) and lighter shades (orange) indicating resistance (higher IC50, lower sensitivity). White cells represent conditions where neutralization was not detected (IC50 > 10 µg/mL).

Since very few compartment-specific differences were identified at sites previously reported to alter bNAb sensitivity, we next directly assessed the functional neutralization sensitivity of viruses from different compartments. To do this, a subset of *n* = 6 participants was selected who displayed a range of genetic divergence between pre-therapy PL and reservoir sequences, and recombinant viruses encoding *env* sequences were generated from pre-therapy PL, PBMCs, and LNs. Partial Env resequencing of a subset of recombinant virus stocks demonstrated that each virus remained highly similar to the corresponding bulk Env PCR product used for virus generation, with phylogenetic analysis confirming close clustering of each virus stock with its parental sequence (Fig. S1). These viruses were tested against a panel of nine bNAbs targeting the CD4-binding site, V2 apex, V3 glycan supersite, membrane proximal external region (MPER), and gp120/gp41 interface using a micro-neutralization TZM-bl-based assay. The resulting neutralization sensitivity is shown as IC50 values in Fig. 3. Representative neutralization curves from two independent experiments for one recombinant virus tested against the full panel of nine bNAbs are shown in Fig. S2.

A central finding of the neutralization analysis was the absence of a consistent pattern of compartment-specific differences in neutralization sensitivity. In many cases, IC50 values were similar across compartments for a given bNAb, while in others, reservoir-derived viruses showed either increased or decreased IC50 values relative to the pre-therapy PL. Overall, paired analyses (Wilcoxon rank signed test) showed no statistically significant differences in neutralization sensitivity between compartments for any bNAb.

It was notable that, overall, most viruses were sensitive to the CD4-binding site antibodies regardless of compartment (IC50 median = 0.11 µg/mL; IQR = 0.05–0.17 µg/mL). Similarly, all viruses, except one (>20 µg/mL), were sensitive to the interface antibody (median = 0.1 µg/mL; IQR = 0.07–0.18 µg/mL). On the other hand, resistance to V2 apex and V3 glycan supersite antibodies was more common and varied between compartments, with IC50s > 10 µg/mL in 80% (CAP256-VRC26.25), 13.3% (PGDM1400), 73.3% (PGT121), and 80% (10-1074) of the viruses. Most viruses were sensitive to the MPER (10E8v4) antibody, except those obtained from participants 651 and 093, which showed partial or complete resistance.

Although this study was not designed to define specific genetic determinants of neutralization sensitivity, we documented the presence of non-consensus mutations at positions previously associated with altered bnAb sensitivity in the sequences included in the neutralization assays (Table S3). In some cases, resistance to the V3-glycan antibodies PGT121 and 10-1074 (e.g., PIDs 3830 and 0098) was accompanied by loss of the N332 glycan motif, a well-described escape mechanism for these antibodies (16), indicating a potential genotype-phenotype relationship. However, this relationship was not consistent. Not all N332-disrupted viruses exhibited high-level resistance, and other sequence differences did not reliably explain variation in neutralization sensitivity across compartments or participants. For example, loss of CAP256-VRC26.25 sensitivity in V2 apex-targeted responses (e.g., PIDs 3830 and 651 reservoir-derived viruses post-ART) occurred without the emergence of additional mutations at known escape sites. Comparable discordance was observed for V3-glycan antibodies in these samples, as well as for PGT151 sensitivity in PID 093 PBMC-derived virus. Overall, these observations suggest that resistance-associated mutations did not reliably predict phenotype, highlighting the importance of phenotypic assays for assessing neutralization sensitivity.

## DISCUSSION

This study investigated whether HIV-1 Env variants from post-therapy reservoirs differ genetically or phenotypically from pre-therapy plasma viruses with respect to bNAb sensitivity. Env sequences from plasma, PBMC, and lymph node compartments clustered by participant, confirming a common origin. Although reservoir sequences contained a number of unique amino acid mutations, few occurred at positions previously associated with altered bNAb sensitivity. Consistent with this observation, functional analyses revealed no consistent compartment-specific differences in neutralization sensitivity between plasma and reservoir-derived viruses. Instead, neutralization profiles were highly variable and participant-specific.

We observed measurable genetic divergence between compartments, with a median of 6 and 19 amino acid differences between pre-therapy PL and LN and PBMC reservoirs, respectively. Next-generation sequencing revealed that a substantial proportion of these mutations were unique to the reservoir and not detected as minor variants in pre-therapy PL. These unique mutations could arise from pre-ART compartmentalization, where distinct viral populations are seeded in different tissues early in infection (7), or low-level ongoing replication during ART, particularly in pharmacologically privileged sites, such as LNs (17, 18). While our data cannot distinguish between these mechanisms, they confirm that the reservoir harbors variants distinct from the pre-ART virus population. Once established, these genetically distinct reservoir variants may be maintained through clonal expansion of infected cells, a mechanism underlying reservoir persistence during suppressive ART. However, only a tiny minority of these unique reservoir mutations occurred at amino acid positions previously reported to alter bNAb sensitivity. This finding is consistent with the absence of bNAb selective pressure in this cohort and suggests that the reservoir may instead be shaped by other factors, such as cytotoxic T lymphocyte (CTL) pressure or fitness constraints imposed by the cellular environment of latently infected cells (19).

A key finding of this study is the absence of consistent compartment-specific patterns in neutralization sensitivity—reservoir viruses may have similar, increased, or decreased sensitivity to bNAbs with respect to the pretherapy plasma virus depending on the participant. Similarly, colleagues report the lack of a uniform direction of differences in neutralization sensitivity between viruses derived from plasma, PBMCs, and lymph nodes in untreated individuals (K. Velan, N. Gumede, S. Mtanjana, N. Zikhali, T. Ndung’u, K. Gounder, and B. Ndlovu, unpublished data). Our findings are also broadly consistent with a computational study by Wang et al. (8), which reported comparable computationally predicted neutralization sensitivity across several different tissue compartments in treated individuals, with the exception of the CNS compartment in some individuals where there was altered resistance for some antibodies. However, our study differs in that we measured phenotypic sensitivity using functional recombinant viruses encoding participant-derived subtype C Env.

The neutralization data observed here revealed clear antibody-class-specific patterns consistent with known structural and functional constraints. Viruses from all compartments and participants were generally sensitive to CD4bs bNAbs (VRC01, VRC07-523LS, N6) and the gp120-gp41 interface bNAb PGT151. This is mechanistically expected, as the CD4bs must engage the host CD4 receptor with high affinity, imposing strong functional constraints that limit mutational escape and make this region a conserved and reliable target for bNAbs (20, 21). Similarly, the gp120-gp41 interface, targeted by PGT151, is involved in essential structural interactions that maintain trimer integrity and support fusion progression, imposing functional constraints on variation (22). In contrast, we observed widespread and variable resistance to bNAbs targeting the V2 apex (CAP256-VRC26.25, PGDM1400) and V3-glycan supersite (PGT121, 10-1074). This variability is characteristic of these epitope classes, which are highly glycosylated, conformationally flexible, and subject to immune pressure (16, 23). The V2 apex is particularly sensitive to glycan shifts (e.g., at N156 or N160) and conformational masking, which can disrupt bNAb recognition (24, 25). Importantly, with the exception of VRC07-523LS, no single bNAb in our panel neutralized all viruses from all compartments at <1 µg/mL, reinforcing the critical need for bNAb combinations targeting non-overlapping epitopes to prevent neutralization escape and achieve broad coverage (26). The broad and consistent sensitivity of reservoir-derived viruses to CD4bs (e.g., VRC07-523LS) and interface (PGT151) bNAbs, aligning with prior studies reporting high sensitivity of subtype C viruses to VRC07-523LS and PGT151 (10, 13) (Velan et al., submitted), suggests that these classes should form core components of combination therapy for subtype C. Their conserved, functional targets minimize the risk of pre-existing resistance within the reservoir. In contrast, the variable sensitivity to V2 apex and V3 glycan supersite antibodies indicates that these may be better deployed as complementary antibodies within combinations.

The high level of resistance to CAP256-VRC26.25 observed in our study (80% of viruses) appears more pronounced than the neutralization breadth reported for this antibody against subtype C viruses in recent studies (10, 13). One possible explanation is methodological differences in virus platforms. Our study employed replication-competent recombinant viruses generated via homologous recombination in an NL4-3 backbone and amplified in T-cell lines, whereas many neutralization data sets are generated using Env-pseudotyped viruses (PSVs) (27). Neutralization outcomes can differ between platforms due to variations in Env density, glycan processing, and spike conformation (26). Replication-competent virions produced from T-cells may more closely approximate native Env organization than PSVs, which can exhibit heterogeneous Env incorporation (28). Sensitivity to bNAbs targeting quaternary epitopes, such as the V2 apex, is particularly influenced by Env density and spatial arrangement, potentially contributing to the higher resistance levels observed here (23, 29).

Several limitations of this study should be considered. The sample size, while typical for intensive tissue-based reservoir studies, limits the generalizability of our findings. Our use of bulk-amplified *env* sequences captures the dominant variants from each compartment, potentially masking the phenotype of minor but rebound-competent reservoir viruses. Additionally, we examined only PBMCs and a single LN, whereas the viral reservoir is distributed across multiple anatomical sites, including the gut and CNS, which may harbor distinct viral populations. The use of a laboratory-adapted NL4-3 backbone for recombinant virus generation, while standard, may not fully recapitulate Env presentation compared with patient-matched backbones.

In summary, the lack of consistent compartment-specific differences in neutralization sensitivity is encouraging for bNAb coverage of the reservoir from different compartments, and results underscore the suitability of the CD4bs and interface as promising therapeutic targets for bNAb therapy. Overall, this work contributes to our understanding of the HIV-1 reservoir in subtype C infection and provides insights that may inform future bNAb-based strategies to control or clear the viral reservoir.

## Data Availability

HIV-1 Env sequences generated in this study are available in GenBank under accession numbers PZ554690–PZ554719. Fastq files from next-generation sequencing are available under BioProject accession number PRJNA1477810 in the NCBI Sequence Read Archive (SRA) database.

## SUPPLEMENTAL MATERIAL

Figure S1 jvi.01151-26-s0001.pdf Phylogenetic tree comparing recombinant virus stock Env sequences with the corresponding bulk Env PCR products used for virus generation.

Figure S2 jvi.01151-26-s0002.pdf Representative neutralization curves.

Table S1 jvi.01151-26-s0003.xlsx HIV-1 Env mutations previously associated with altered neutralization sensitivity.

Table S2 jvi.01151-26-s0004.xlsx Next-generation sequencing (NGS) data at Env positions known to alter bNAb sensitivity that differ between matched pre-treatment plasma-derived sequences and LN/PBMC-derived HIV sequences.

Table S3 jvi.01151-26-s0005.xlsx Summary of non-consensus amino acids at HIV-1 Env positions known to affect sensitivity to bNAbs in phenotyped participants.

ASM does not own the copyrights to Supplemental Material that may be linked to, or accessed through, an article. The authors have granted ASM a non-exclusive, world-wide license to publish the Supplemental Material files. Please contact the corresponding author directly for reuse.
