# Supplementary material for: Genetic divergence between reservoir and pre-therapy plasma HIV-1 Env variants does not confer consistent compartment-specific differences in broadly neutralizing antibody sensitivity
Source: J Virol. 2026 Sep 11;100(10):e01151-26. doi: 10.1128/jvi.01151-26 (PMC13712996; doi:10.1128/jvi.01151-26)
Supplement: Supplementary file 1 [file jvi.01151-26-s0001.pdf]

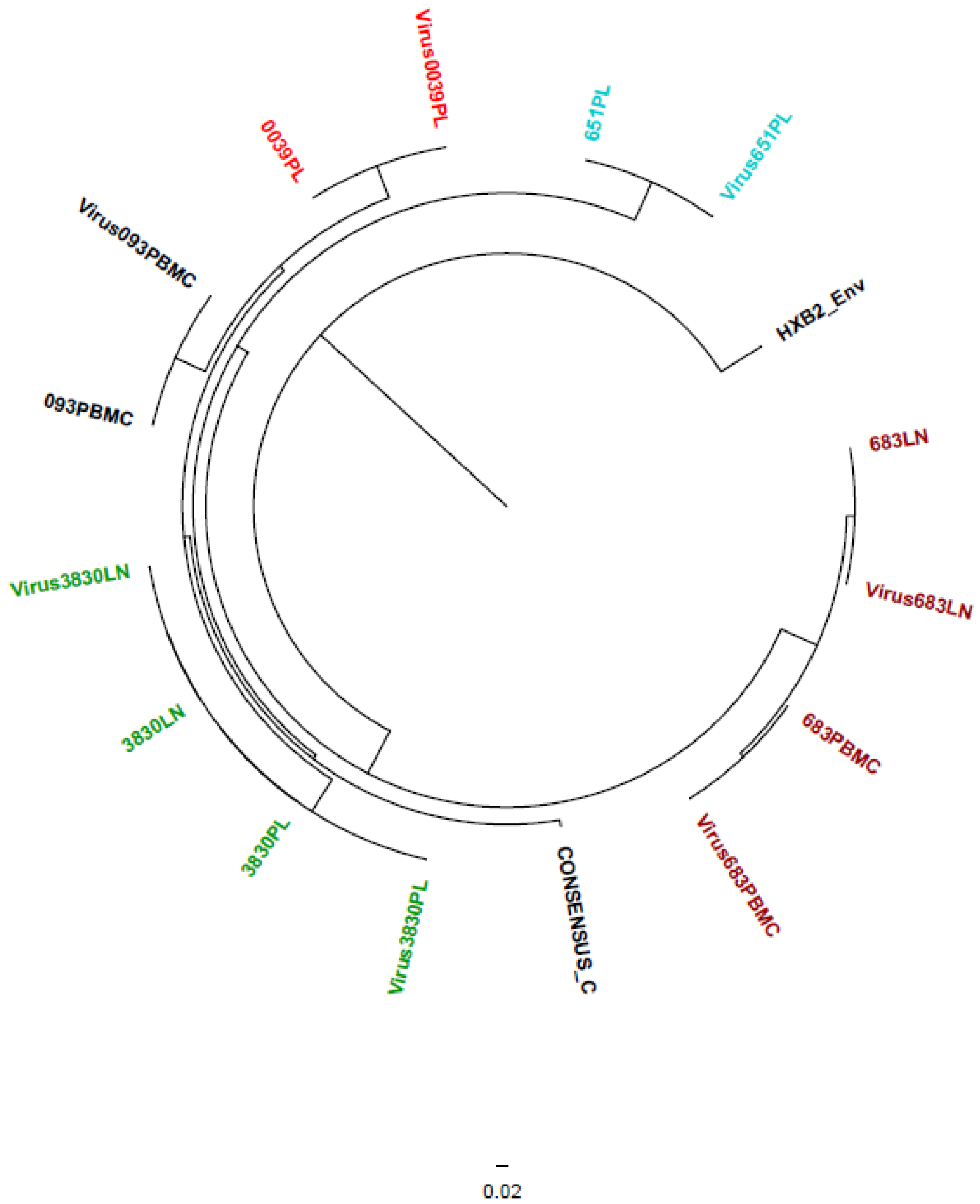

Supplementary Figure 1. Verification of recombinant virus stock Env sequences.

A maximum-likelihood phylogenetic tree comparing partial Env sequences (237–286 amino acids) obtained from recombinant virus stocks with the corresponding bulk Env PCR products used for virus generation. Each recombinant virus clustered with its corresponding PCR product.
