# Supplementary material for: Genetic divergence between reservoir and pre-therapy plasma HIV-1 Env variants does not confer consistent compartment-specific differences in broadly neutralizing antibody sensitivity
Source: J Virol. 2026 Sep 11;100(10):e01151-26. doi: 10.1128/jvi.01151-26 (PMC13712996; doi:10.1128/jvi.01151-26)
Supplement: Supplementary file 2 [file jvi.01151-26-s0002.pdf]

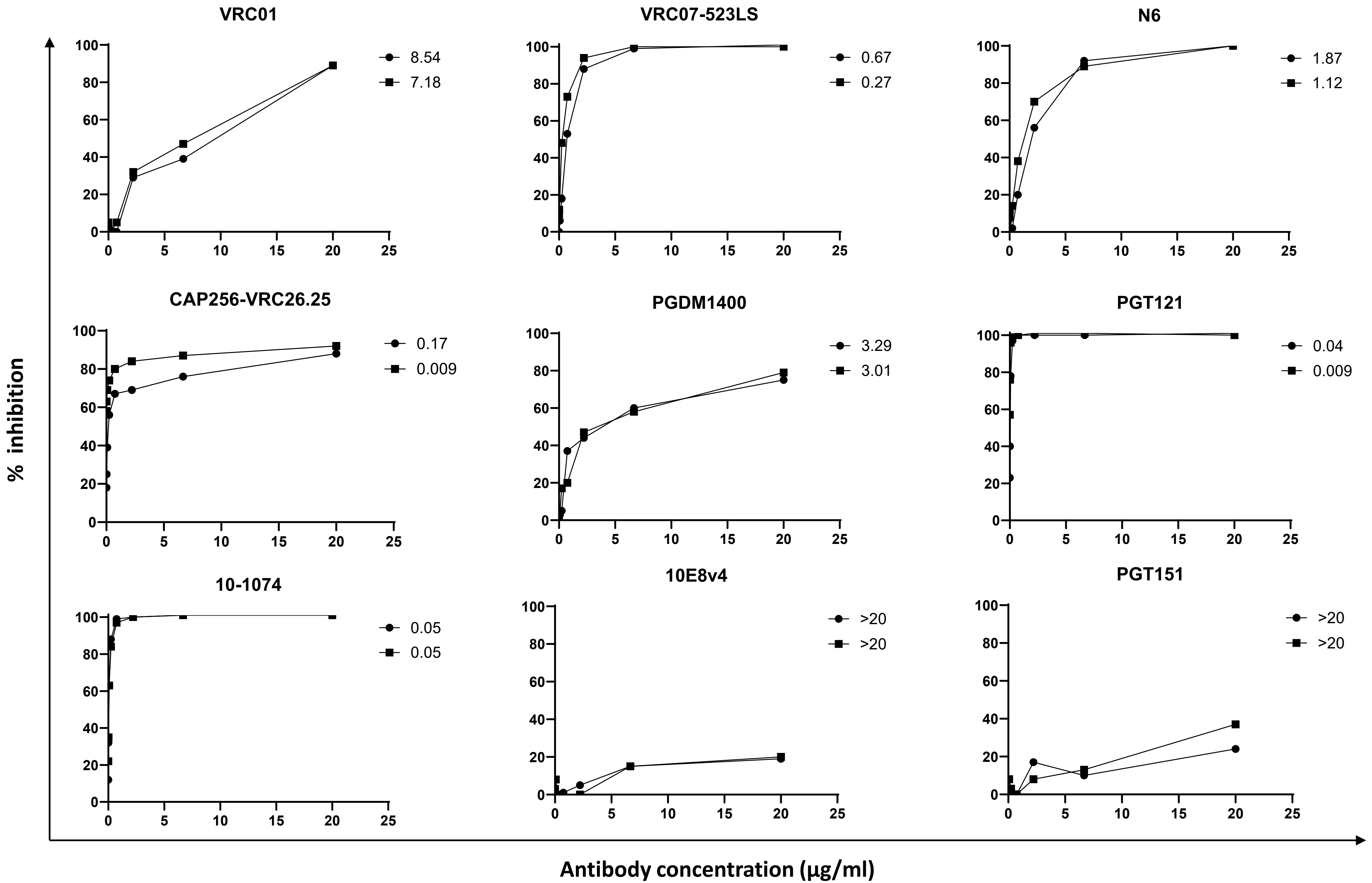

VRC01
8.54
7.18
VRC07-523LS
0.67
0.27
N6
1.87
1.12
CAP256-VRC26.25
0.17
0.009
PGDM1400
3.29
3.01
PGT121
0.04
0.009
10-1074
0.05
0.05
10E8v4
>20
>20
PGT151
>20
>20
% inhibition
Antibody concentration (µg/ml)

Supplementary Figure 2. Neutralization curves.

Representative neutralization curves for one recombinant virus (PID 093 PBMC) tested against the panel of nine broadly neutralizing antibodies. Curves from two independent experiments are shown. IC50 values for each experiment are indicated. The IC50 values reported in the manuscript were derived from replicate neutralization assays performed for all viruses.
